# Exposure of Zero-Dose Children to Multiple Deprivation: Analyses of Data from 80 Low- and Middle-Income Countries

**DOI:** 10.3390/vaccines10091568

**Published:** 2022-09-19

**Authors:** Andrea Wendt, Thiago M. Santos, Bianca O. Cata-Preta, Luisa Arroyave, Daniel R. Hogan, Tewodaj Mengistu, Aluisio J. D. Barros, Cesar G. Victora

**Affiliations:** 1 International Center for Equity in Health, Federal University of Pelotas, Rua Marechal Deodoro 1160, Pelotas 96020-220, RS, Brazil; 2 Programa de Pós-Graduação em Tecnologia em Saúde, Pontifícia Universidade Católica do Paraná, Rua Imaculada Conceição 1155, Curitiba 80215-901, PR, Brazil; 3 Gavi, the Vaccine Alliance, Chemin du Pommier 40, 1218 Geneva, Switzerland

**Keywords:** drinking water, education, family planning, immunization, inequality, malaria, sanitation, stunting, vaccination, wasting

## Abstract

The concept of multiple deprivation recognizes that the same individuals, households, and communities are often exposed to several forms of scarcity. We assessed whether lack of immunization is also associated with nutritional, environmental, and educational outcomes. We analyzed data from nationally representative surveys from 80 low- and middle-income countries with information on no-DPT (children aged 12–23 months without any doses of a diphtheria, pertussis and tetanus containing vaccine), stunting, wasting, maternal education and use of contraception, improved water and sanitation, and long-lasting insecticidal nets. Analyses of how these characteristics overlap were performed at individual and ecological levels. Principal component analyses (PCA) provided additional information on indicator clustering. In virtually all analyses, no-DPT children were significantly more likely to be exposed to the other markers for deprivation. The strongest, most consistent associations were found with maternal education, water, and sanitation, while the weakest associations were found for wasting and bed nets. No-DPT prevalence reached 46.1% in the most deprived quintile from first PCA component derived from deprivation indicators. All children were immunized in the two least deprived quintiles of the component. Our analyses provide strong support for the hypothesis that unimmunized children are also affected by other forms of deprivation.

## 1. Introduction

The 17 Sustainable Development Goals (SDGs) comprise an ambitious agenda with multisectoral goals covering health, nutrition, water and sanitation, education, and several other topics, with the ultimate aim of achieving “a better and more sustainable future for all” [1]. Child immunization contributes to the achievement of the SDGs and is specifically mentioned in SDG 3.8 on universal health coverage. The Immunization Agenda 2030 (IA2030) launched in 2020, outlines the global vision and strategy for achieving immunization for all [2].

Despite substantial progress in scaling up coverage of newer vaccines over the past decade, increasing coverage of basic routine immunizations beyond 80% globally has proven more challenging, even more so given disruptions related to the COVID-19 pandemic [3]. Reaching missed children and communities with immunization requires new strategies, and recent studies have suggested that integrated approaches should be considered [4,5]. Immunization, as well as several other major health services, have been reliant on vertical programs in many low and middle-income countries (LMICs), with separate management structures and funding from other health interventions as well as programs directed at other sectors. More recently, there have been calls for integrating immunization programs with broader primary health care and universal coverage strategies and initiatives [2,6]. There is also renewed interest in going beyond the health sector by integrating immunization and other child health services with the delivery of other programs in education, child protection, nutrition, water, sanitation, and hygiene [7].

The concept of multiple deprivation recognizes that the same individuals, households, and communities are often exposed to several forms of scarcity. Calls for integration are consistent with attempts to quantify multiple deprivation in LMICs using summary indices. The Global Multidimensional Poverty Index (MDPI) [8,9,10] is calculated on the basis of 10 indicators, one of which is child mortality. Building upon this approach, UNICEF has proposed the Multiple Overlapping Deprivation Analysis (MODA) methodology based on up to 70 indicators derived from national surveys, one of which is “incomplete vaccination (e.g., child has not received BCG and all three DPT vaccinations by age 23 months)” [11]. Both MDPI and MODA focus on individuals who are being simultaneously affected by more than one type of deprivation, and their results have been used to estimate the numbers of individuals affected by poverty in specific geographies.

A complementary approach to multidimensional indices has been the study of how different types of deprivation overlap at the individual or household level. In the context of women’s and children’s health, the concept of co-coverage was introduced in 2005 to assess how many health interventions have been received by each child and his/her mother [12]. Similar analyses on the overlap among different interventions have been recently carried out for studying how zero-dose children differ from immunized children in terms of coverage with antenatal care, institutional delivery, careseeking for common childhood illnesses, and access to handwashing facilities in the home [5].

Integrated approaches to service delivery can be efficient ways to reduce inequalities in health service coverage because children and households that are missing out on immunization are often also being missed by other health interventions. In the present article, we look beyond the health sector to characterize a fuller range of opportunities for integration. Our goal is to investigate whether zero-dose children, i.e., those missing out on routine vaccination, and their families are missing out on other health services and interventions in relevant sectors such as nutrition, environmental health, and education. We look at how vaccination coverage is associated with child undernutrition, household access to safe water and sanitation, maternal education, use of family planning services and insecticide-treated bed nets for malaria prevention. Our results on multideprivation may help build the evidence base for multisectoral strategies aimed at identifying missed communities and planning pro-equity strategies for delivery of immunization and other essential services.

## 2. Materials and Methods

### 2.1. Sample and Study Design

The International Center for Equity in Health (ICEH) database includes over 450 nationally representative surveys—mostly Demographic and Health Surveys (DHS, https://dhsprogram.com/, accessed on 1 August 2022) and Multiple Indicator Cluster Surveys (MICS, https://mics.unicef.org/, accessed on 1 August 2022)—which provide information on households, women of reproductive health (15–49 years) and children under the age of five years. Both families of surveys use standardized data collection procedures, making the results comparable across surveys and countries. More detailed information about DHS and MICS can be found elsewhere [13,14].

We have analyzed individual data from the most recent surveys carried out since 2010 in LMICs with complete information on all indicators required for the analyses. If more than one survey was available after 2010, we selected the most recent one. As is the praxis for immunization analyses, the sample was restricted to children aged 12–23 months for most countries, except in countries where measles vaccine is delivered after 12 months of age, namely Egypt and Bosnia and Herzegovina (children aged 18–29 months) and Turkey and Moldova (children aged 15–26 months). Although the measles vaccine is not included the present analyses, we used these age ranges to ensure comparability with previous studies and survey reports [15,16].

### 2.2. Immunization Indicator

Given the 2030 goal of achieving immunization for all and reaching zero-dose children (that is, those who fail to receive any routine vaccinations) we focus on no-DPT prevalence, defined as the proportion of children who did not receive any doses of a DPT (diphtheria, pertussis, and tetanus)-containing vaccine, including tetravalent and pentavalent vaccines. No-DPT has been adopted as the operational measure for zero-dose children by the immunization community [17,18,19] and has the advantage of being estimable from routine health information systems, and not only from survey data. In the present context, we refer to no-DPT children as a synonym for zero-dose children.

### 2.3. Multiple Deprivation Indicators

To measure multiple deprivation, we selected a set of seven indicators for social, environmental, or nutritional disadvantage. The indicators were selected on the basis of data availability, and their definitions comply with the Countdown to 2030 initiative for monitoring the SDGs relevant to women [20], children, and adolescents, as follows:

- Stunting: proportion of children with a length-for-age z-score below 2 standard deviations relative to the median of the WHO Child Growth Standards [21].
- Wasting: proportion of children with a weight-for-length z-score below 2 standard deviations relative to the median of the WHO Child Growth Standards.
- Lack of improved water: proportion of children living in a household without improved water source. The following sources were considered as improved: piped water into dwelling, plot, or yard; piped water into neighbor’s plot; public tap/standpipe; tubewell/borehole; protected dug well; protected spring; rainwater. Packaged water: bottled, sachet and refill water.
- Lack of improved sanitation: proportion of children living in a household without improved sanitation. The following facilities were considered as improved: sewage is disposed of in a sanitary way (flush or pour-flush to a piped sewer system, a septic tank, or a pit latrine; ventilated improved pit latrine (VIP); pit latrine with slab; composting toilet). Shared sanitary facilities are not considered as improved.
- Lack of long-lasting insecticidal net (LLIN): proportion of children living in a household without a LLIN. This was restricted to malaria-endemic countries with data on LLIN.
- Mother without education: proportion of children whose mothers had no education.
- Mother without demand for family planning satisfied (mDFPS): proportion of children whose mothers (in union and in need of contraception) are using a modern contraceptive method.

### 2.4. Statistical Analysis

Two related sets of analyses were carried out, at the individual level (households, women, and children), and at ecological level (country and subnational areas).

### 2.5. Individual Level Analyses

For the individual level, we considered the child as the unit of analysis. In households with multiple children, all were included. We assessed associations between no-DPT status and the presence of stunting or wasting in the child, household environmental characteristics (presence of bed nets, improved water, and sanitation), maternal education and family planning. Prevalence ratios (PRs) were estimated dividing the prevalence of each of the seven deprivation indicators in no-DPT children by the same prevalence in vaccinated children. The chi-squared test for heterogeneity was used to assess statistical significance of the associations, and 95% confidence intervals (CIs) were calculated for the PRs.

### 2.6. Ecological Analyses

The ecological analyses assessed the associations of no-DPT prevalence and the seven deprivation indicators at two levels of aggregation: countries and subnational areas. Results include Spearman correlation coefficients and beta coefficients (β) from estimated linear regressions.

### 2.7. Additional Analyses

Lastly, we carried out principal component analyses (PCA, which is a data reduction method) to check whether our eight variables are related among themselves and could be summarized by a single continuous index of deprivation. This index is the first component, which explains the largest proportion of the variability in the dataset. We then examined how strongly each of the eight indicators was associated with the first component, in order to assess their contributions to an overall index. The units of analysis were all children in the 80 countries, weighted by national child population sizes. Using the PCA results we created a deprivation index in quintiles, ranging from least to most deprived. We also estimated the prevalence of each indicator according to deprivation index quintiles. These analyses are presented in the supplementary materials.

We used Stata 17.0 Stata (StataCorp. 2019. Stata Statistical Software: Release 17. College Station, TX: StataCorp LLC) and R (R Core Team, 2020, version 4.1.3. R Foundation for Statistical Computing, Vienna, Austria) for all analyses and figures taking in account the complex sampling. Pooled estimates were weighted by the populations of children 12–23 months in each country obtained from World Bank databases [22]. Additional information about sample weight adjustment for population of children in each country is presented in the Supplementary materials.

## 3. Results

The analyses included data from 80 LMICs with surveys carried out in 2010 or later, with information on immunizations, stunting, wasting, maternal education, mDFPS, improved water and improved sanitation, and long-lasting insecticidal nets. Our sample included 57% of all LMICs (74% of all low-income, 66% of all lower-middle-, and 39% of all upper-middle-income countries), considering the World Bank income classification in 2016 the median year of the surveys. The 80 countries include 61% of all children aged 12–23 months living in LMICs. Several LMICs with large child populations were not included because either they lacked standardized surveys (e.g., China and Brazil) or because their surveys lacked one or more indicators required for the analyses (e.g., Indonesia, Ethiopia, the Philippines, and Vietnam).

We present individual-level analyses based on a total of 177,266 children, followed by ecological analyses at country level for the 80 countries and at subnational-area level for 827 regions (445 with information on LLIN). The full list of countries and information about the surveys is available in Supplementary Tables S1 and S2.

Table 1 shows the pooled analyses including all children from the 80 countries. Results were weighted using national child population sizes. The prevalence of each deprivation indicator was calculated separately for vaccinated and unvaccinated children, and the ratios and differences between each set of two prevalence measures are shown. In general, the prevalence of deprivation indicators was higher in no-DPT children compared to those vaccinated. As shown in Table 1, the strongest associations were observed for lack of improved water and for mothers with no education. Lack of access to improved water was 32.8% among no-DPT children compared to 12.6% among those who had been vaccinated. The corresponding prevalence levels for lack of maternal education were 53.4% and 23.5%. All pooled ratios were statistically significant, except for lack of LLIN, which was similarly distributed in both groups.

**Figure 1:**
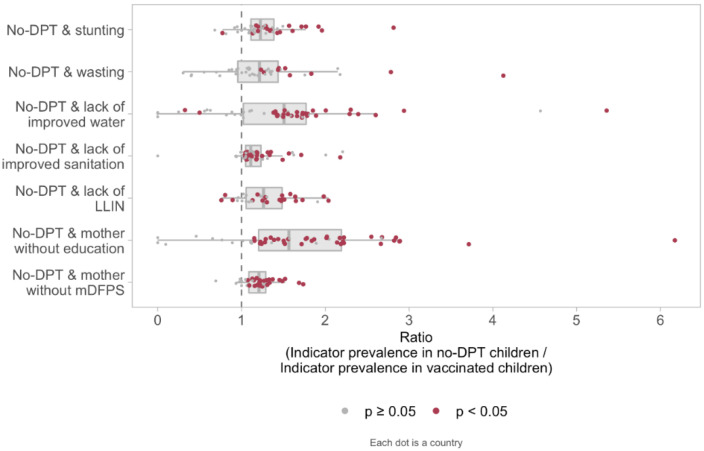
Distribution of no-DPT prevalence ratios according to deprivation indicators in the individual-level analyses in 80 countries (39 countries for bed nets). Each dot represents one country and red dots indicate PRs for which the 95% CI did not include unity. Note: The extreme left of box represents the 25th percentile and the extreme right the 75th. The vertical line within the box represents the median. The whiskers extend to the largest or smallest value no further than 1.5 times the interquartile range. The medians and interquartile ranges are calculated with every country having the same weight. Legend: DPT: diphtheria, pertussis, tetanus. LLIN: long-lasting insecticidal nets. mDFPS: demand for family planning satisfied with modern methods.

Figure 1 shows the variability among countries in terms of the PRs of deprivation indicators according to vaccination status, based on all children from each country. Each dot represents one country and red dots indicate PRs for which the 95% CI did not include unity. For most countries, no-DPT prevalence was higher among children exposed to other deprivation indicators, particularly in terms of lack of improved water and mothers with no education. Yet, results varied considerably from country to country.

**Figure 2:**
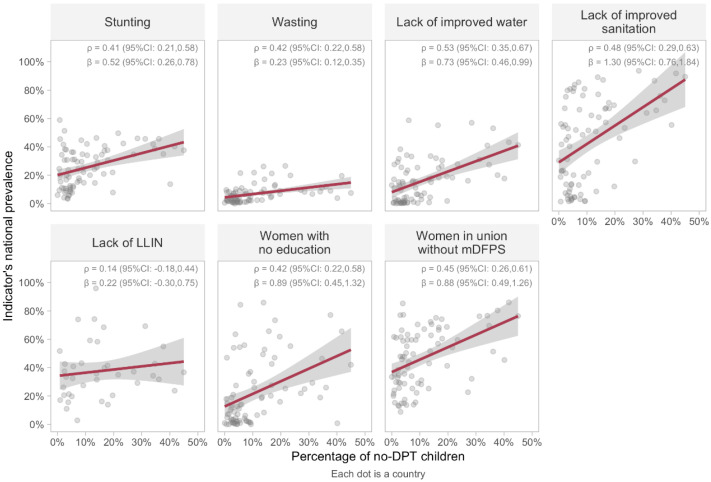
Associations between no-DPT prevalence and prevalence of deprivation indicators in 80 countries (39 countries for bed nets). Linear regression lines are shown in red, and 95% CI in grey. ρ: Spearman correlation coefficient. Legend: DPT: diphtheria, pertussis, tetanus. LLIN: long-lasting insecticidal nets. mDFPS: demand for family planning satisfied with modern methods. CI: confidence interval.

Figure 2 shows the results of ecological analyses using countries as the units of analysis, with no-DPT prevalence in the *x*-axes. The strongest correlation coefficients were observed for lack of improved water (ρ = 0.53; 95%CI: 0.35–0.67), followed by lack of improved sanitation (ρ = 0.48; 95%CI: 0.29–0.63) and women without family planning needs satisfied (ρ = 0.45; 95%CI: 0.26–0.61). The weakest correlation was observed for lack of LLIN (ρ = 0.14; 95%CI: −0.18–0.44). The linear regressions betas (β) were all positive. The highest beta was observed for lack of sanitation, with each increase of one percentage point in no-DPT prevalence being associated with an increase of 1.3 percentage points in the prevalence of lack of sanitation.

**Figure 3:**
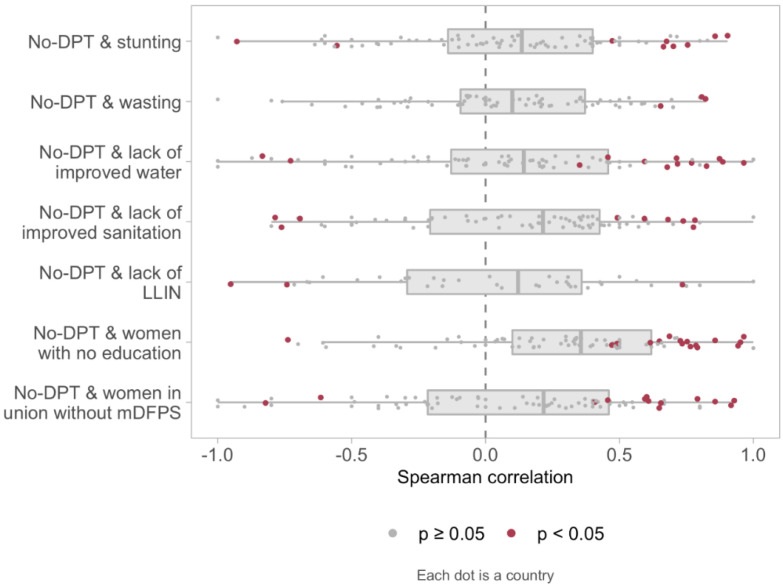
Distribution of correlation coefficients between no-DPT prevalence and deprivation indicators at country level in 80 countries (39 countries for bed nets). Each dot represents one country and red dots indicate PRs for which the 95% CI did not include the unity. Note: The extreme left of box represents the 25th percentile and the extreme right the 75th. The vertical line within the box represents the median. The whiskers extend to the largest or smallest value no further than 1.5 times the interquartile range. The medians and interquartile ranges are calculated with every country having the same weight. Legend: DPT: diphtheria, pertussis, tetanus. LLIN: long-lasting insecticidal nets. mDFPS: demand for family planning satisfied with modern methods.

Figure 3 shows the distribution of Spearman correlation coefficients between no-DPT and deprivation indicators at country level. Each dot represents the coefficient in a country. There is a marked variability, but for most countries, the correlation coefficients are positive and statistically significant.

**Figure 4:**
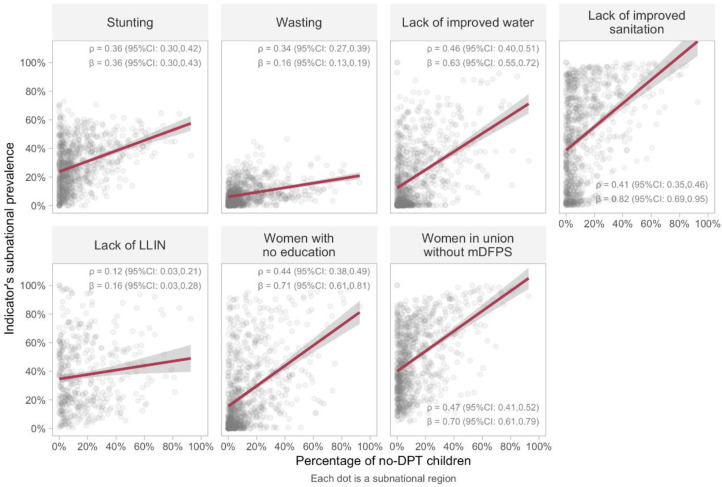
Associations between no-DPT prevalence and prevalence of deprivation indicators in 827 subnational areas (445 for bed nets). Linear regression lines are shown in red, and 95%CI in grey. ρ: Spearman correlation coefficient. Legend: DPT: diphtheria, pertussis, tetanus. LLIN: long-lasting insecticidal nets. mDFPS: demand for family planning satisfied with modern methods. CI: confidence interval.

The results of country-level analyses were replicated using subnational areas (Figure 4). Both sets of results were similar to the country level analysis. In this case, the highest correlation with no-DPT prevalence was found for lack of family planning (ρ = 0.47; 95%CI: 0.41–0.52), followed by lack of improved water (ρ = 0.46; 95%CI: 0.40–0.50) and by women with no education (ρ = 0.44; 95%CI: 0.38–0.49). As in the country level analyses, lack of bed nets was weakly correlated with immunization, and the highest beta (0.82) was observed for lack of sanitation. Use of non-linear models did not improve the correlations.

**Figure 5:**
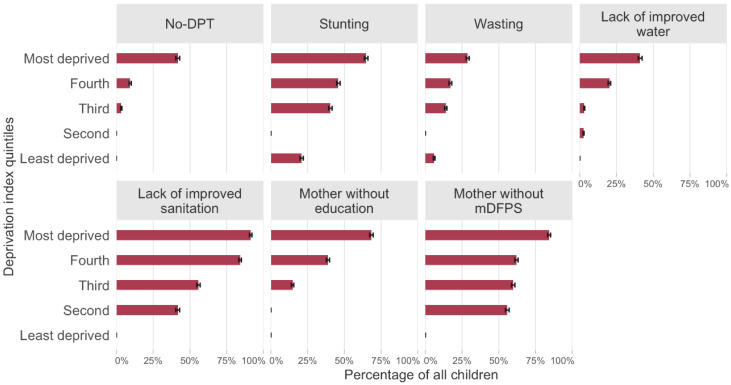
Prevalence of each indicator according to deprivation index quintiles (all 80 countries). Legend: DPT: diphtheria, pertussis, tetanus. mDFPS: demand for family planning satisfied with modern methods.

Finally, the PCA results are shown in Table 2 and Figure 5. The first component explained 29.1% of the variance. Loadings were all positive indicating that all deprivation indicators including no-DPT prevalence are positively associated. The highest loadings in the analyses including the 80 countries were observed for indicators of sanitation, maternal education, and contraception. Figure 5 shows the prevalence of each indicator included in the PCA, according to quintiles of the first PCA component. All outcome indicators are clearly associated with the summary variable. For example, no-DPT prevalence reached 41.6% in the most deprived quintile, whereas all children had been immunized in the two least deprived quintiles. When we included the LLIN indicator for countries with this information (39 countries), the PCA was very similar. Supplementary Figure S1 shows similar results for the 39 countries with information on LLINs, and Supplementary Figure S2 shows the results of a PCA model without the immunization indicator.

## 4. Discussion

Building upon previous assessments of co-coverage of health interventions [5,12], our present analyses expand the scope to investigate overlaps among immunization and seven indicators of multiple deprivation in terms of nutrition, education, and the environment. Using data on 177,266 children from 80 LMICs with 827 subnational areas, we examined associations with no-DPT prevalence. We found significant positive associations in nearly all analyses, showing that children who had not been immunized were also at higher risk of presenting with other deprivation indicators. The strongest, most consistent associations across the individual-level and ecological analyses were found for maternal education, water, and sanitation deprivation indicators. The weakest associations were found for wasting and bed nets, whereas associations with stunting and family planning tended to be moderate. Our findings from the principal component analyses confirm that the same children, households, and communities tend to be exposed to several forms of multiple deprivation. Our results are consistent with existing multiple deprivation indices [8,9,10,11].

The analyses of within-country inequalities in child immunization coverage traditionally rely on household asset indices and maternal education as stratification variables and report important gaps in most countries. Of the three indicators most strongly associated with no-DPT prevalence in the present analysis, one is maternal education and the other two–water and sanitation–are part of household asset indices [23]. Our results are therefore compatible with the literature, in addition to providing a more granular examination of which specific deprivation indicators tend to be more closely associated with immunization [24].

Our analysis also points to substantial variability in deprivation patterns across countries, suggesting that efforts to tackle multiple deprivation should be tailored to the epidemiological and socio-economic context of each country. Doing this will likely require the triangulation of different data sources, some of which may be collected by different government ministries or organizations. One potential data source that may be worth considering anew for immunization-focused planning and analysis are national censuses. Information on immunization coverage is seldom collected in national censuses, but these routinely obtain data on deprivation indicators on water, sanitation, and education [25,26]. Our findings of an overlap among multiple dimensions of deprivation suggests that census data that can be disaggregated for small geographic units may be useful for indicating communities where zero-dose children are most likely to be found.

The strengths of our findings include the large number of countries covered, the use of three sets of units of analyses (individuals, subnational regions, and countries), and the estimation of standardized indicators based on the original survey datasets. We also summarized the different deprivation indicators in a single continuous index of deprivation using PCA. This index had clear associations with the individual outcomes. Our analyses also have limitations. The 80 countries studied represent 57.1% of all LMICs, but upper-middle-income countries are underrepresented because of lack of standardized surveys in many of them, including China and Brazil. Information on vaccines is mostly derived from inspection of vaccine cards, but for 23% of the children in the sample no cards were available in the home and the information was based on respondent’s recall. For indicators based on household data (water, sanitation, and LLIN), children in our age range living in the same household were assigned the same information. Only 3.4% of all households had more than one child in this age range. An additional limitation is that surveys included in the analyses took place over a ten-year period, and the situation may have changed in recent years, for better or for worse, particularly in light of the COVID-19 pandemic’s effect on health services.

## 5. Conclusions

Our results provide empirical evidence and quantify the degree to which zero-dose children and their families face multiple deprivations, which are apparent at individual, subnational, and national levels. Targeting zero-dose children will likely target missed communities who could benefit from other health services as well as better nutrition, education, and water and sanitation. An example of a complementary intervention was the application of the Reach Every District strategy in Byanzurkh District in Mongolia. It included a wider package of maternal, newborn, and child health services, and also environmental health, water, and sanitation [27]. In our principal component analysis, the vast majority of zero-dose children were classified as being in the most deprived quintile of the deprivation index. More generally, our results underscore the potential impact of multisectoral interventions to improve equity in health and development outcomes on the pathway to achieving the Sustainable Development Goals.

## Figures and Tables

**Table 1:** Prevalence of deprivation indicators according to vaccination status. Individual-level analyses pooled over 80 countries.

Deprivation Indicator	Vaccination Status	% Deprived Children	Ratio	Difference	*p*-Value	N of Children	N of Countries
Stunting	No-DPT	43.9%	1.32	10.6	<0.001	147,614	80
	Vaccinated	33.3%					
Wasting	No-DPT	15.2%	1.20	2.6	<0.001	147,593	80
	Vaccinated	12.6%					
Lack of improved water	No-DPT	32.8%	2.60	20.2	<0.001	177,687	80
	Vaccinated	12.6%					
Lack of improved sanitation	No-DPT	71.2%	1.35	18.4	<0.001	177,687	80
	Vaccinated	52.8%					
Lack of LLIN	No-DPT	45.2%	1.02	0.8	0.076	79,229	39
	Vaccinated	44.4%					
Mother without education	No-DPT	53.4%	2.27	29.9	<0.001	177,650	80
	Vaccinated	23.5%					
Mother without mDFPS	No-DPT	69.3%	1.42	20.4	<0.001	116,545	80
	Vaccinated	48.9%					

Legend: DPT: diphtheria, pertussis, tetanus. LLIN: long-lasting insecticidal nets. mDFPS: demand for family planning satisfied with modern methods.

**Table 2:** Principal component analysis loadings for the eight deprivation indicators. Pooled analyses based on all children from the 80 countries (39 countries for bed nets).

Indicator	Loadings	
	All 80 Countries	39 Countries with LLIN Information
No-DPT	0.31	0.32
Stunting	0.38	0.36
Wasting	0.24	0.19
Lack of improved water	0.29	0.32
Lack of improved sanitation	0.50	0.45
Mother without education	0.43	0.35
Mother without mDFPS	0.43	0.45
Lack of LLIN	--	0.33

Legend: DPT: diphtheria, pertussis, tetanus. LLIN: long-lasting insecticidal nets. mDFPS: demand for family planning satisfied with modern methods.

## Data Availability

All the analyses were carried out using publicly available datasets that can be obtained directly from the DHS (dhsprogram.com) the MICS (mics.unicef.org) websites. Datasets are continuously sourced and updated by the International Center for Equity in Health (equidade.org) as they are released. We used the last available versions in 13 May 2022.

## Supplementary Materials

The following supporting information can be downloaded at: https://www.mdpi.com/article/10.3390/vaccines10091568/s1, Section SA: Sample weight adjustments for population 12–23 months. Section SB: Table S1: Number of children with information on DPT and each multiple deprivation indicator for each survey included in the analysis at individual level. Table S2: Surveys included in ecological analysis. Figure S1: Prevalence of each indicator according to deprivation index quintiles (countries with information on LLIN). No-DPT was one of the variables used in the PCA. Figure S2: Prevalence of no-DPT according to deprivation index quintiles (all countries). No-DPT was not included in the PCA.
