# Supplementary material for: Exposure of Zero-Dose Children to Multiple Deprivation: Analyses of Data from 80 Low- and Middle-Income Countries
Source: Vaccines (Basel). 2022 Sep 19;10(9):1568. doi: 10.3390/vaccines10091568 (PMC9502633; doi:10.3390/vaccines10091568)
Supplement: Supplementary file 1 [file vaccines-10-01568-s001.zip › vaccines-1883966-supplementary.pdf]

Supplementary material

Section A

Sample weight adjustments for population 12-23 months

In order to take into consideration the number of children aged 12-23 months living in each country, individual sample weights were adjusted using the following equation:

$$w_{ij,adj} = \left(\frac{w_{ij}}{\sum_i w_{ij}}\right)\left(\frac{pop_j}{\sum_j pop_j}\right)N$$

where:

i indicates a child and *j* a country

$w_{ij,adj}$ is the adjusted individual sample weight

w_{ij} is the original sample weight

pop_j is the population of children aged 12-23 months in the country *j*

N is the total number of children in the sample

Section B

Table S1. Number of children with information on DPT and each multiple deprivation indicator for each survey included in the analysis at individual level.

Country	Year	Source	Stunting	Wasting	Lack of improved water	Lack of improved sanitation	Lack of LLIN	Mother without education	Mother without mDFPS
Algeria	2018	MICS	2780	2793	2953	2953	-	2953	2436
Angola	2015	DHS	1369	1374	2845	2845	2845	2845	1285
Armenia	2015	DHS	318	317	345	345	-	345	297
Bangladesh	2017	DHS	1599	1607	1666	1666	-	1666	1549
Belize	2015	MICS	478	476	503	503	-	498	382
Benin	2017	DHS	2465	2466	2522	2522	2522	2522	1632
Bosnia and Herzegovina	2011	MICS	477	464	516	516	-	516	412
Burkina Faso	2010	DHS	1368	1356	2791	2791	2791	2791	1670
Burundi	2016	DHS	1274	1278	2596	2596	2596	2596	1696
CAR	2018	MICS	1644	1653	1688	1688	1688	1688	882
Cambodia	2014	DHS	933	935	1441	1441	-	1441	1230
Cameroon	2018	DHS	926	924	1824	1824	1824	1824	918
Chad	2014	DHS	1811	1826	2880	2880	1917	2880	1079
Comoros	2012	DHS	532	533	630	630	630	629	448
Congo Brazzaville	2014	MICS	1686	1703	1773	1773	1773	1772	861
Congo Democratic Republic	2017	MICS	4138	4190	4251	4251	4251	4250	2133
Costa Rica	2018	MICS	635	633	708	708	-	708	445
Côte d'Ivoire	2016	MICS	1768	1774	1784	1784	1784	1784	934
Cuba	2019	MICS	1118	1116	1119	1119	-	1119	771
Egypt	2014	DHS	3047	3032	3204	3204	-	3204	2426
El Salvador	2014	MICS	1485	1482	1504	1504	-	1504	1053
Eswatini	2014	MICS	531	531	540	540	-	540	221
Gabon	2012	DHS	745	750	1197	1197	1197	1197	690
Gambia	2018	MICS	1870	1871	1895	1895	1895	1890	1151
Ghana	2017	MICS	1665	1664	1681	1681	1681	1681	1095

Guatemala	2014	DHS	2354	2360	2408	2408	-	2408	1823
Guinea	2018	DHS	716	706	1408	1408	1408	1408	680
Guinea Bissau	2018	MICS	1392	1398	1409	1409	1409	1407	625
Guyana	2014	MICS	619	617	688	688	-	687	512
Haiti	2016	DHS	1132	1126	1196	1196	1196	1196	964
Honduras	2011	DHS	2172	2172	2277	2277	-	2277	1725
India	2015	DHS	46593	46410	49284	49284	-	49284	35260
Iraq	2018	MICS	3159	3161	3205	3205	-	3205	2394
Kazakhstan	2015	MICS	1053	1061	1103	1103	-	1103	789
Kenya	2014	DHS	3871	3884	4052	4052	4051	4052	1365
Kiribati	2018	MICS	447	446	453	453	-	452	284
Kosovo	2019	MICS	214	215	294	294	-	294	228
Kyrgyzstan	2018	MICS	623	624	643	643	-	643	410
Lao	2017	MICS	2140	2162	2215	2215	2215	2215	1641
Lesotho	2018	MICS	649	649	667	667	-	667	426
Liberia	2019	DHS	517	520	1063	1063	1063	1063	593
Madagascar	2018	MICS	2521	2528	2590	2590	2590	2590	1490
Malawi	2015	DHS	1055	1067	3248	3248	3248	3248	2507
Maldives	2016	DHS	471	470	590	590	-	590	504
Mali	2018	DHS	1821	1827	1946	1946	1946	1946	1072
Mauritania	2015	MICS	2020	2020	2131	2131	2131	2126	1374
Mexico	2015	MICS	1501	1495	1536	1536	-	1534	1099
Moldova	2012	MICS	345	343	386	386	-	386	283
Mongolia	2018	MICS	1054	1049	1077	1077	-	1077	781
Montenegro	2013	MICS	252	251	266	266	-	266	162
Myanmar	2015	DHS	850	845	915	915	915	915	734
Namibia	2013	DHS	406	400	991	991	991	991	409
Nepal	2019	MICS	1247	1296	1327	1327	-	1325	1149
Niger	2012	DHS	902	907	2151	2151	2151	2146	1117
Nigeria	2018	DHS	2391	2400	6059	6059	6059	6059	2766
North Macedonia	2018	MICS	247	247	307	307	-	307	243

Pakistan	2017	DHS	674	678	1893	1893	1891	1893	1278
Papua New Guinea	2016	DHS	670	663	1816	1816	1816	1816	1371
Paraguay	2016	MICS	974	977	1012	1012	-	1012	721
Rwanda	2014	DHS	732	734	1537	1537	1537	1537	1095
São Tomé and Príncipe	2019	MICS	343	341	349	349	349	348	223
Senegal	2019	DHS	1142	1137	1183	1183	1183	1183	796
Serbia	2019	MICS	236	235	384	384	-	384	272
Sierra Leone	2019	DHS	873	877	1861	1861	1861	1861	1032
South Africa	2016	DHS	225	208	670	670	-	670	248
State of Palestine	2019	MICS	1205	1197	1330	1330	-	1330	953
Sudan	2014	MICS	2396	2384	2641	2641	-	2638	1331
Suriname	2018	MICS	634	637	763	763	-	751	505
Tajikistan	2017	DHS	1267	1263	1297	1297	-	1297	776
Tanzania	2015	DHS	2075	2080	2158	2158	2158	2158	1419
Thailand	2019	MICS	2638	2631	2879	2879	-	2879	1790
Timor-Leste	2016	DHS	1229	1220	1423	1423	1423	1423	895
Togo	2017	MICS	969	968	973	973	973	973	682
Tonga	2019	MICS	231	230	246	246	-	245	122
Tunisia	2018	MICS	632	635	656	656	-	656	561
Turkey	2013	DHS	584	579	764	764	-	764	680
Turkmenistan	2015	MICS	772	773	787	787	-	787	528
Uganda	2016	DHS	932	931	2922	2922	2922	2922	2009
Yemen	2013	DHS	2801	2818	3053	3053	-	3053	2499
Zambia	2018	DHS	1840	1849	1928	1928	1928	1928	1335

Legend: DPT: Diphtheria, Pertussis, Tetanus. LLIN: long-lasting insecticidal nets. mDFPS: demand for family planning satisfied with modern methods.

Table S2. Surveys included in ecological analysis.

Country	Year	Source	No-DPT children	Stunted children	Wasted children	Household members without improved water	Household members without improved sanitation	Household members without LLIN	Mother without education	Women without mDFPS
Algeria	2018	MICS	2953	2780	2793	29919	29919	-	14873	11718
Angola	2015	DHS	2845	1369	1374	16072	16072	16072	14322	3849
Armenia	2015	DHS	345	318	317	7893	7893	-	1724	2761
Bangladesh	2017	DHS	1666	1599	1607	19457	19457	-	8759	13986
Belize	2015	MICS	503	478	476	4636	4636	-	2515	2344
Benin	2017	DHS	2522	2465	2466	14156	14156	14156	13589	5350
Bosnia and Herzegovina	2011	MICS	516	477	464	5778	5778	-	2297	2013
Burkina Faso	2010	DHS	2791	1391	1379	14415	14415	14415	15039	5445
Burundi	2016	DHS	2596	1274	1278	15976	15976	15976	13192	5572
Cambodia	2014	DHS	1441	1062	1065	15821	15821	-	7165	7926
Cameroon	2018	DHS	1824	926	924	11693	11693	11693	9733	3323
CAR	2018	MICS	1688	1644	1653	8133	8133	8133	8921	3144
Chad	2014	DHS	2880	1880	1899	17229	17229	11380	18623	3612
Comoros	2012	DHS	630	586	586	4474	4474	4474	3138	1723
Congo Brazzaville	2014	MICS	1773	1686	1703	12811	12811	12811	9167	3293
Congo Democratic Republic	2017	MICS	4251	4138	4190	20792	20792	20792	21455	6431
Costa Rica	2018	MICS	708	635	633	8490	8490	-	3612	3444
Côte d'Ivoire	2016	MICS	1784	1768	1774	11879	11879	11879	9094	3227
Cuba	2019	MICS	1119	1118	1116	11966	11966	-	5254	4823
Egypt	2014	DHS	3204	3077	3057	28162	28162	-	15848	14288
El Salvador	2014	MICS	1504	1485	1482	12507	12507	-	7339	6390
Eswatini	2014	MICS	540	531	531	4865	4865	-	2688	1453
Gabon	2012	DHS	1197	864	868	9733	9733	9733	6067	2687
Gambia	2018	MICS	1895	1870	1871	7405	7405	7405	9891	3891

Country	Year	Source	No-DPT children	Stunted children	Wasted children	Household members without improved water	Household members without improved sanitation	Household members without LLIN	Mother without education	Women without mDFPS
Ghana	2017	MICS	1681	1665	1664	12886	12886	12886	8879	4552
Guatemala	2014	DHS	2408	2428	2434	21373	21373	-	12440	11116
Guinea	2018	DHS	1408	716	706	7912	7912	7912	7951	2510
Guinea Bissau	2018	MICS	1409	1392	1398	7379	7379	7379	7482	2524
Guyana	2014	MICS	688	619	617	5077	5077	-	3353	2648
Haiti	2016	DHS	1196	1132	1126	13405	13405	13405	6530	5511
Honduras	2011	DHS	2277	2323	2323	21349	21349	-	10888	10925
India	2015	DHS	49284	47722	47538	601137	601137	-	259627	323291
Iraq	2018	MICS	3205	3159	3161	20214	20214	-	16623	11859
Kazakhstan	2015	MICS	1103	1053	1061	16500	16500	-	5510	5107
Kenya	2014	DHS	4052	4112	4128	36405	36405	36402	20964	6450
Kiribati	2018	MICS	453	447	446	3071	3071	-	2175	1462
Kosovo	2019	MICS	294	214	215	5124	5124	-	1539	2065
Kyrgyzstan	2018	MICS	643	623	624	6968	6968	-	3546	2238
Lao	2017	MICS	2215	2140	2162	22287	22287	22287	11719	12479
Lesotho	2018	MICS	667	649	649	8847	8847	-	3256	2652
Liberia	2019	DHS	1063	517	520	9062	9062	9062	5704	2787
Madagascar	2018	MICS	2590	2521	2528	17870	17870	17870	12851	6315
Malawi	2015	DHS	3248	1055	1067	26337	26337	26337	17286	12436
Maldives	2016	DHS	590	471	470	6044	6044	-	3106	2915
Mali	2018	DHS	1946	1821	1827	9507	9507	9507	9940	3186
Mauritania	2015	MICS	2131	2020	2020	11765	11765	11765	10641	4662
Mexico	2015	MICS	1536	1501	1495	10760	10760	-	8063	6397
Moldova	2012	MICS	386	345	343	11354	11354	-	1869	2519
Mongolia	2018	MICS	1077	1054	1049	13798	13798	-	6091	5112
Montenegro	2013	MICS	266	252	251	4052	4052	-	1420	911

Country	Year	Source	No-DPT children	Stunted children	Wasted children	Household members without improved water	Household members without improved sanitation	Household members without LLIN	Mother without education	Women without mDFPS
Myanmar	2015	DHS	915	921	915	12500	12500	12500	4815	5213
Namibia	2013	DHS	991	532	526	9826	9826	9826	5046	2510
Nepal	2019	MICS	1327	1247	1296	12655	12655	-	6655	7870
Niger	2012	DHS	2151	946	956	10749	10749	10749	12537	3113
Nigeria	2018	DHS	6059	2391	2400	40392	40392	40392	33924	10219
North Macedonia	2018	MICS	307	247	247	4082	4082	-	1506	1537
Pakistan	2017	DHS	1893	674	678	11869	11869	11866	10473	5996
Papua New Guinea	2016	DHS	1816	670	663	16021	16021	16012	9514	6565
Paraguay	2016	MICS	1012	974	977	7313	7313	-	4625	3757
Rwanda	2014	DHS	1537	761	765	12698	12698	12698	7856	4980
São Tomé and Príncipe	2019	MICS	349	343	341	3426	3426	3426	1839	1274
Senegal	2019	DHS	1183	1142	1137	4538	4538	4538	6125	2788
Serbia	2019	MICS	384	236	235	6346	6346	-	1838	1777
Sierra Leone	2019	DHS	1861	873	877	13399	13399	13399	9899	4403
South Africa	2016	DHS	670	225	208	11066	11066	-	3548	1953
State of Palestine	2019	MICS	1330	1205	1197	9326	9326	-	6328	4448
Sudan	2014	MICS	2641	2396	2384	16801	16801	-	14065	4223
Suriname	2018	MICS	763	634	637	7915	7915	-	4227	3571
Tajikistan	2017	DHS	1297	1267	1263	7840	7840	-	6195	3924
Tanzania	2015	DHS	2158	2075	2080	12563	12563	12563	10233	4802
Thailand	2019	MICS	2879	2638	2631	35604	35604	-	13684	14500
Timor-Leste	2016	DHS	1423	1229	1220	11498	11498	11498	7221	3818
Togo	2017	MICS	973	969	968	7916	7916	7916	4941	2630
Tonga	2019	MICS	246	231	230	2498	2498	-	1346	750
Tunisia	2018	MICS	656	632	635	11225	11225	-	3420	4336
Turkey	2013	DHS	764	584	579	11794	11794	-	3648	5369

Country	Year	Source	No-DPT children	Stunted children	Wasted children	Household members without improved water	Household members without improved sanitation	Household members without LLIN	Mother without education	Women without mDFPS
Turkmenistan	2015	MICS	787	772	773	5861	5861	-	3765	2953
Uganda	2016	DHS	2922	932	931	19588	19588	19588	15522	7556
Yemen	2013	DHS	3053	2869	2886	17346	17346	-	16093	9623
Zambia	2018	DHS	1928	1840	1849	12829	12829	12829	9959	5211

Legend: DPT: Diphtheria, Pertussis, Tetanus. LLIN: long-lasting insecticidal nets. mDFPS: demand for family planning satisfied with modern methods.

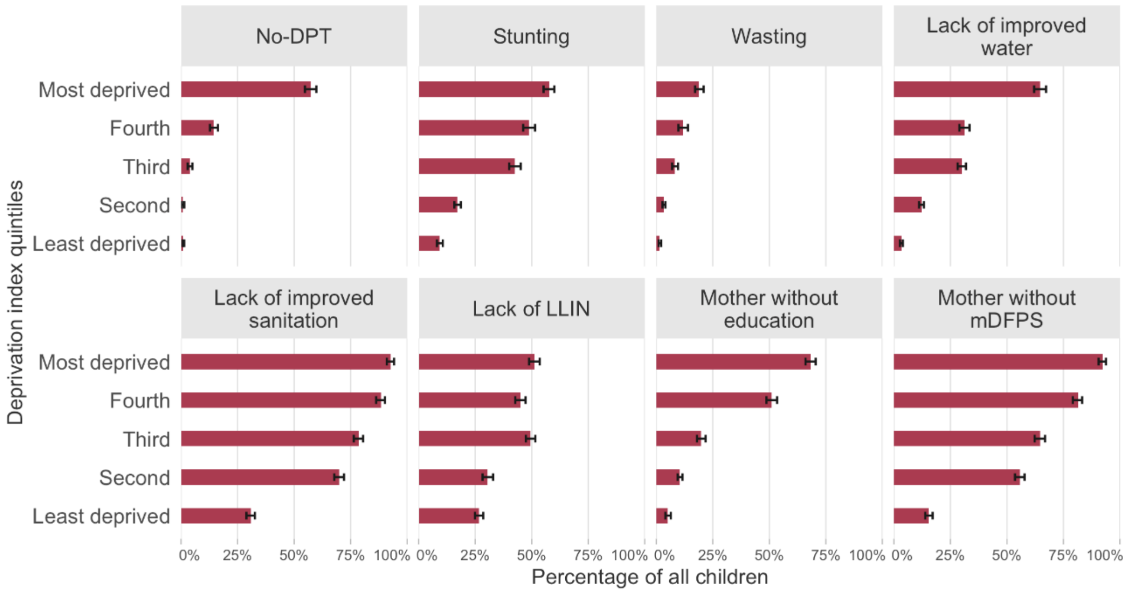

Figure S1. Prevalence of each indicator according to deprivation index quintiles (countries with information on LLIN). No-DPT was one of the variables used in the PCA. Legend: DPT: Diphtheria, Pertussis, Tetanus. LLIN: long-lasting insecticidal nets. mDFPS: demand for family planning satisfied with modern methods. PCA: principal component analysis.

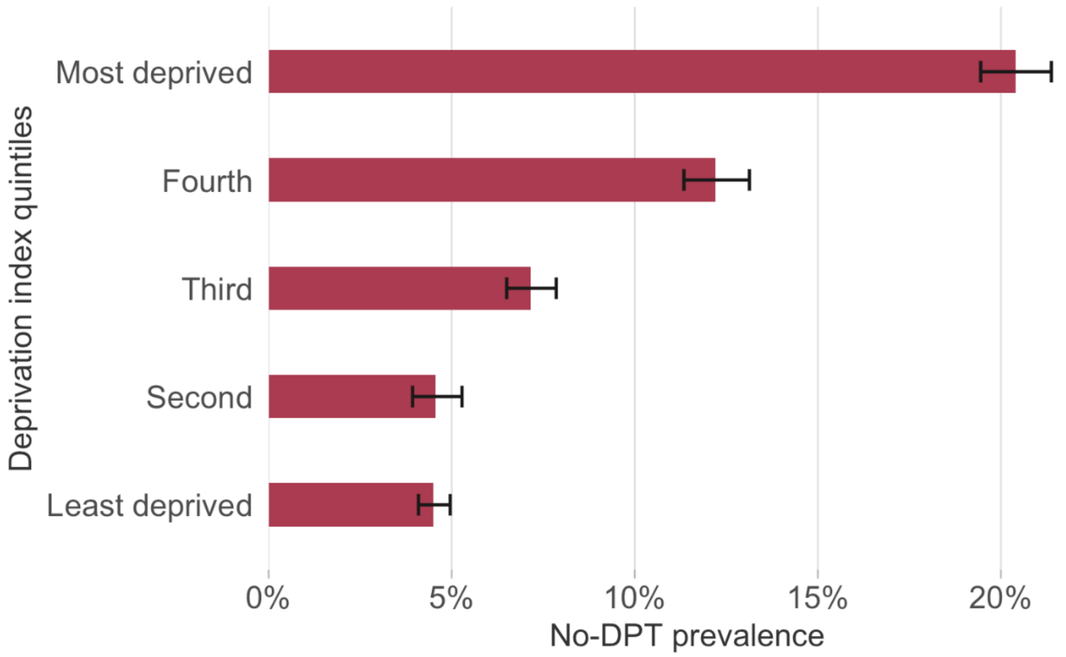

Figure S2. Prevalence of no-DPT according to deprivation index quintiles (all countries. No-DPT was not included in the PCA. Legend: DPT: Diphtheria, Pertussis, Tetanus. PCA: principal component analysis.
